# Quality of Life in Internet Use Disorder Patients With and Without Comorbid Mental Disorders

**DOI:** 10.3389/fpsyt.2022.862208

**Published:** 2022-03-24

**Authors:** Jan Dieris-Hirche, Bert Theodor te Wildt, Magdalena Pape, Laura Bottel, Toni Steinbüchel, Henrik Kessler, Stephan Herpertz

**Affiliations:** ^1^Department of Psychosomatic Medicine and Psychotherapy, LWL-University Hospital, Ruhr University Bochum, Bochum, Germany; ^2^Psychosomatic Hospital Diessen Monastery, Dießen am Ammersee, Germany

**Keywords:** internet use disorder, internet addiction, gaming disorder, quality of life, comorbid, dual diagnosis, behavioral addiction

## Abstract

**Introduction:**

Evidence from clinical studies on quality of life (QoL) in patients suffering from internet use disorders (IUD) is still limited. Furthermore, the impact of additional mental comorbidities on QoL in IUD patients has rarely been investigated yet.

**Materials and Methods:**

In a cross-sectional clinical study 149 male subjects were analyzed for the presence and severity of an IUD as well as other mental disorders by experienced clinicians. The sample consisted of 60 IUD patients *with* and *without* comorbid mental disorders, 34 non-IUD patients with other mental disorders, and 55 healthy participants. Standardized clinical interviews (M.I.N.I. 6.0.0) and questionnaires on IUD symptom severity (s-IAT), QoL (WHOQOL-BREF), depression and anxiety symptoms (BDI-II and BAI), and general psychological symptoms (BSI) were used.

**Results:**

Internet use disorder patients showed significantly reduced QoL compared to healthy controls (Cohen’s *d* = 1.64–1.97). Furthermore, IUD patients suffering from comorbid mental disorders showed significantly decreased levels of physical, social, and environmental QoL compared to IUD patients without any comorbidity (*p* < 0.05–0.001). Multiple linear regression analyses revealed that low levels of psychological, social and environmental QoL were mainly predicted by symptoms of depression. IUD factors were only significant predictors for the social and physical QoL.

**Discussion:**

Internet use disorder patients with comorbid mental disorder reported the lowest QoL. Depression symptom severity was the most significant predictor of low QoL in IUD. Strategies to reduce depressive symptoms should therefore be considered in IUD treatment to increase patients’ QoL.

## Introduction

Internet use disorder (IUD) is a general term defined as an excessive and uncontrolled use of Internet applications in terms of an online behavioral addiction. It includes both excessive online-gaming (as the largest category) and non-gaming internet activities, i.e., online shopping, pornography use, social network use, and general Internet use (1). Consistent with the inclusion of (Internet) Gaming Disorder (IGD) as the first IUD in the ICD-11 (2), many researchers switched from using the term Internet addiction to IUD to be in accordance with the terminology used in the upcoming ICD-11 (1).

Internet use disorder has increased in the last decades with worldwide prevalence rates ranging between 2.6% in northern and western Europe and 10.9% in the Middle East; the average global prevalence is estimated at a level of 6.0% (3). In German populations, the IUD prevalence rates range between 1.2 and 3.1% (4–8). However, most studies of IUD prevalence were conducted using screening questionnaires, which increases the risk of false positives or high prevalence. Consequently, studies on clinical samples using standardized diagnostic examinations by experts are significant, although the samples are often smaller.

Individuals with IUD show a persistent pattern of Internet use that is characterized by impaired control regarding the onset, intensity and duration of usage (2). The increased priority given to Internet activities leads to neglect of daily activities and life interests, and IUD is associated with social, physical and mental burden (9, 10). In addition, high comorbidity with psychiatric disorder has been reported, especially depressive disorders, anxiety disorders, attention deficit hyperactive disorder, substance use disorders and impulse control disorders (11–16). Although representative studies often report a balanced gender distribution (5, 6, 17, 18), it is predominantly male IUD patients who come to clinics for therapy (19, 20).

So far, quality of life (QoL) in problematic or pathological Internet use was examined in studies surveyed in non-clinical contexts, such as high school, college students, or in the general population (21–25). Evidence from studies of QoL in IUD patients in a clinical context is still limited (26). The comparison of QoL between IUD patients and patients with other mental disorders has only been considered in online surveys, but not in clinical populations (27). Hence, the current study investigates the impairment of QoL in a clinical group of male IUD patients using high methodological standards, in particular standardized clinical interviews by experienced clinicians.

The research questions (RQ) and hypotheses (H) were:

(1) RQ1: Is there any impairment at all in QoL domains of patients with IUD (compared to healthy control group)? H1: We expected that IUD patients show lower levels of QoL compared to healthy controls.
(2) RQ2: If so, how do IUD patients *with* comorbid mental disorders differ in QoL from IUD patients *without* comorbid mental disorders? H2: We expect greater QoL impairment in IUD patients *with* comorbidities.
(3) RQ3: Considering IUD patients, what factors predict low levels of QoL in different domains? H3: We expect that IUD factors are significant predictors of QoL.
(4) RQ4: Do IUD patients’ QoL differ from patients with other (non-IUD) mental disorders? If so, in which way?

## Materials and Methods

### Participants and Procedure

The trial was conducted as a cross-sectional study on men only. The limitation to male IUD patients resulted from the fact that IUD treatment seekers were almost exclusively men. Between 2016 and 2017 a total of *n* = 60 male IUD patients according to DSM-5 criteria (experimental group, EG) were recruited in an IUD specialized outpatient clinic at a university hospital in Germany. All patients were individuals who came to the clinic on their own initiative to seek diagnosis and treatment. The patients were examined for the presence of an IUD as well as other mental disorders by experienced clinicians using standardized clinical interviews. In total, 31 IUD patients *without* other comorbid mental disorders (EG1) and 29 IUD patients *with* at least one further comorbid mental disorder (EG2) were recruited and diagnosed. Furthermore, two age-matched male control groups (CG1 and CG2) were recruited. CG1 was 1:1 matched with the EG, consisted of 57 healthy male participants, and was recruited *via* an advertisement in local newspapers. CG2 was 1:1 matched to EG1 and EG2, consisted of 34 male non-IUD patients with different mental disorders (CG2), and were also recruited at the same outpatient clinic in Germany. In order to obtain standardized information about the mental disorder present, both control groups were also examined using the same diagnostic procedure. Thus, a total of 151 subjects were finally included in the study. The inclusion criteria were: legal age, mastery of the German language, presence of an IUD (EG1 and EG2), presence (CG2) or absence (CG1) of other mental disorders. The exclusion criteria for EG and CG2 were: psychosis or delusional disorder, bipolar disorder, complex post-traumatic stress disorder and acute substance addiction (without tobacco). These mental disorders were excluded as they were assumed to be so severe, acute or complex and thus lead to a confounding of the effects of the study. The exclusion criterium for CG1 was the presence of any mental disorder. In accordance with the Helsinki Declaration of 1975 as revised in 1983, the Ethics Committee of the Ruhr-University Bochum approved the study (*registration number*: 15-5155). All participants were informed about the study prior to participation and provided written informed consent.

### Measures

#### Structured Diagnosis of Mental Disorders

Mental disorders were diagnosed by the clinical neuropsychiatric interview M.I.N.I. adapted to the German language, version 6.0.0 (28). The M.I.N.I. interview is based on the international classification systems ICD-10 and DSM-4. In addition, a structured clinical interview was conducted for diagnosing IUD that was based on the research criteria for online computer game addiction according to DSM-5 (29). These criteria have been adapted for different types of IUD (e.g., cybersex, social media, online streaming). IUD was diagnosed if at least five of the nine DSM-5-criteria were present over a 12-month period.

#### Internet Addiction Behavior

The *Short version of the Internet Addiction Test* (s-IAT) (30) was used to measure the IUD symptom severity. The self-report questionnaire consists of 12 items, which are rated on a 5-point-Likert scale from 1 (“rarely”) to 5 (“always”) with a total score range from 12 to 60 points. Higher scores reflecting higher IUD symptom severity. Sum scores above the cut-off of 37 points indicate pathological Internet use. For this study, Cronbach’s α = 0.94.

#### Quality of Life

The *World Health Organization’s WHOQOL-BREF* (31, 32) was used to assess quality of life. The WHOQOL-BREF creates four domain scores using 24 items: a 7-item “physical health” domain, a 6-item “psychological health” domain, a 3-item “social relationships” domain, and an 8-item “environment” domain. Transformed domain scores resulted in a 0–100 scale in which higher scores indicate higher QoL. WHOQOL-BREF has been validated in dozens of countries and languages, and among healthy and clinical populations (33). For this study, Cronbach’s α for the domains ranged between 0.60 and 0.83.

#### Depressive Symptoms

The *Beck’s Depressions Inventory, revised* (BDI-II) (34, 35) was used to measure depression symptom severity. It consists of 21 items representing 21 symptoms of depression, which can be rated from 0 to 3 in terms of intensity based on the last 7 days. The total score ranges from 0 to 63, with higher scores indicating higher symptom severity. For this study, Cronbach’s α = 0.94.

#### Anxiety Symptoms

The *Beck Anxiety Inventory* (BAI) measures anxiety with a focus on somatic symptoms of anxiety that was developed to discriminate between anxiety and depression (36). Responses are rated in 21 items on a 4-point Likert scale and range from 0 (not at all) to 3 (severely). The total score ranges from 0 to 63 with higher scores indicating higher symptom severity. For this study, Cronbach’s α = 0.92.

#### Impairment Due to Physical and Psychological Symptoms

The *Brief Symptom Inventory* (BSI) is an abbreviated form of the revised Symptom Check List (SCL-90-R) and includes 53 items to assess psychological symptom burden in adults (37, 38). It includes nine subscales and a global severity score (GSI) that is used to assess basic psychological distress. For this study, Cronbach’s α for the subscales ranged between 0.78 and 0.90.

### Statistical Analysis

Analyses were conducted with IBM SPSS statistics for Macintosh (Version 26.0, Armonk, NY, United States: IBM Corp.) and G*Power for Macintosh, version 3.1 (39). Normal distribution was estimated using Q–Q plots. Bootstrapping method [1,000 samples, bias corrected and accelerated (BCa)] was used in analyses for variables that were obviously not normally distributed. To compare group differences, chi-square distribution for categorical variables (using effect size φ), independent *t*-test for metrically scaled variables (using effect size d) as well as univariate analysis of variance (ANOVA) with Bonferroni post-hoc test (using effect size η^2^) was used. Multiple linear regressions (stepwise) were used to estimate the relationship between multiple independent variables and quality of life domains as dependent variable (using effect size f^2^). Associations between age, IUD symptoms, psychiatric symptoms and quality of life were analyzed using bootstrapped Pearson correlations and partial correlations controlling for confounding variables. The following effect sizes were calculated following Cohen (40): Eta squared η^2^ = 0.01 small, η^2^ = 0.06 moderate, η^2^ = 0.14 large effect. Cohen’s *d* = 0.20 small, *d* = 0.50 moderate, and *d* = 0.80 large effect. Phi-coefficient φ = 0.10 small, φ = 0.30 moderate, φ = 0.50 large effect. Cohen’s f^2^ = 0.02 small, f^2^ = 0.15 moderate, f^2^ = 0.35 large effect.

### Study Size

A recently published study conducted on college students to examine quality of life and Internet addiction showed significant differences between the two groups “with Internet addiction” and “without Internet addiction” with an effect size of *d* = 0.69, 95% CI [0.44, 0.96] (41). An a-priori power analysis was performed for the independent sample *t*-test using this estimated effect size *d* = 0.69. It indicated that a sample of 34 people per group (total *N* = 68) would be needed to reach a power (1 − β error probability) = 0.80 using α = 0.05. A post-hoc *t*-test analysis conducted with our final results (60 IUD patients, 55 healthy controls, effect size for QoL environment *d* = 1.64) indicated a power > 99% (2-sided). Furthermore, a post-hoc power analysis for each linear regression was conducted using *n* = 60 IUD participants, the effect sizes f^2^ [calculated using the formula f^2^ = R^2^/1 – R^2^ (40)], α = 0.05, and nine tested predictors. All these analyses showed a power > 99%.

## Results

### Outliners

Boxplots and histograms were used to identify extreme outliers for the variables BDI, BAI, s-IAT, and all QoL domains of the WHOQOL-BREF. In total, *n* = 2 extreme outliers were excluded from the healthy control group (CG1) due to extreme BDI-2 scores. The final total number of analyzed participants consisted of *n* = 149 subjects, including 31 IUD patients without comorbidity (EG1), 29 IUD patients with comorbidity (EG2), 55 healthy control subjects (CG1) and 34 patients with other mental disorders (CG2).

### Sociodemographic Characteristics and Internet Use Characteristics

Group differences on sociodemographic characteristics, comorbidity, and Internet use characteristics are presented in Table 1.

**TABLE 1:** Group differences on sociodemographic characteristics and internet use disorder (IUD) attributes.

Variables	IUD patients		Healthy controls		Other mental disorders		*p* value	Effect size
			
	(EG1+EG2)		(CG1)		(CG2)			
	*n* = 60		*n* = 55		*n* = 34			
*Age* (*M, SD*)	25.07	5.76	24.38	4.05	27.03	5.41	0.06	η^2^ = 0.038
*BMI* (*kg/m^2^*)	26.92	5.83	24.37	4.06	27.17	7.34	0.023	η^2^ = 0.037
*Marital status* (*n, % within each group*)							<0.001*^b^*	φ = 0.368
Partnership	13	21.70%	33	60.00%	9	26.50%		
Single	47	78.30%	22	40.00%	25	73.50%		
*Education* (*n, % within each group*)							<0.026*^b^*	φ = 0.221
High school graduate	33	55.00%	42	76.40%	18	52.90%		
Less than high school	27	45.00%	13	23.60%	16	47.10%		
*Employment* (*n, % within each group*)							<0.001*^b^*	φ = 0.434
Currently in training/university	25	41.70%	25	45.50%	7	20.60%		
Completed vocational training	9	15.00%	7	12.70%	13	38.30%		
University degree	4	6.70%	14	25.40%	6	17.60%		
No vocational training	22	36.60%	7	12.70%	8	23.50%		
Others	0	0%	2	3.70%	0	0%		
***Time spent on the internet (M, SD)***								
Weekdays (h/d)	6.15	3.47	2.46	1.8	3.35	2.38	<0.001*^c^*	η^2^ = 0.281
At the weekend (h/d)	8.19	4.35	3.38	2.76	4.27	2.98	<0.001*^c^*	η^2^ = 0.287
Average use/day (h/d)	6.74	3.54	2.72	2.03	3.62	2.49	<0.001*^c^*	η^2^ = 0.300
Total time of use per week (h/w)	47.15	24.79	19.09	14.19	25.32	17.49	<0.001*^c^*	η^2^ = 0.300
*IUD subgroups* (*n, %*)								
Gaming	46	76.70%	–	–	–	–		
Cybersex/pornography	9	15.00%	–	–	–	–		
Others/mixed IUD	5	8.30%	–	–	–	–		
*s-IAT score* (*M, SD*)	37.2	10.09	17.42	4.62	20.64	6.75	<0.001*^c^*	η^2^ = 0.591
***Type and frequencies of (*comorbid*) *mental disorders* (*n, % within the group*)^a^***								
Internet Use Disorder	60	100%	–	–	0	0%		
Depressive disorders	19	31.70%	–	–	22	64.7%%	0.002	φ = 0.320
Panic disorder	3	5.00%	–	–	2	5.90%	0.855	φ = 0.019
Agoraphobia	7	11.70%	–	–	3	8.80%	0.668	φ = 0.044
Social phobia	14	23.30%	–	–	2	5.9%%	0.031	φ = 0.223
Generalized anxiety disorder	2	3.30%	–	–	3	8.80%	0.254	φ = 0.118
Obsessive-compulsive disorder	1	1.70%	–	–	0	0%	0.449	φ = 0.078
Anorexia nervosa	0	0%%	–	–	3	8.8%%	0.019	φ = 0.241
Others	1	1.7%%			7	20.0%%	0.002	φ = 0.318
*Mean number of mental disorders incl. IUD*	1.82	1.06	–	–	1.43	0.85	0.031	*d* = 0.99

*One missing value in the group of IUD patients regarding the time of use.*

*^a^In the IUD group, only n = 29 patients showed comorbid mental disorders.*

*^b^Post-hoc tests of Chi^2^ analyses were compared by examining the adjusted residuals. Bonferroni correction for repeated testing was used.*

*^c^Post-hoc tests of ANOVAs were conducted using Bonferroni correction.*

### Comparison of Quality of Life in Internet Use Disorders Patients and Healthy Individuals

The first step was to determine whether patients with IUDs had any impairment in QoL at all (RQ1). Bootstrapped independent-sample *t*-tests indicated significantly decreased QoL in all four domains (Cohen’s *d* = 1.64–1.97) for IUD patients (EG) compared to the healthy controls (CG1) (see Table 2). On the standardized scale between 0 and 100, IUD patients thus indicated a 48.9% decreased QoL in social relations compared to the CG1, followed by the psychological (decreased by 36.2%), physical (decreased by 29.8%), and environmental (decreased by 25.6%) domain. The global difference in QoL across all four domains was 35.1% as compared to the healthy CG1. H1 could thus be confirmed.

**TABLE 2:** QoL domains: IUD patients vs. healthy controls.

QoL domains	IUD patients		Healthy controls		*t*(113)	*p*	Cohen’s *d*
		
	(EG1+EG2), *n* = 60		(CG1), *n* = 55				
		
	M	SD	M	SD			
Physical health	42.92	12.84	61.10	7.36	−9.35	<0.001	1.72
Psychological	44.44	15.27	69.55	9.07	−10.36	<0.001	1.97
Social relationship	39.44	22.17	77.12	15.97	−21.14	<0.001	1.93
Environment	62.92	15.44	84.49	10.08	−29.75	<0.001	1.64

*Mean parameter values for each of the analyses are shown for IUD patients (n = 60) and healthy controls (n = 55), as well as the results of bootstrapped t tests (assuming unequal variance) comparing the QoL domains.*

### Role of Mental Comorbidity in Comparison of Quality of Life

After analyzing that IUD patients generally showed a reduction in QoL, the group was divided into EG1 (*n* = 31 IUD patients *without* other mental comorbidity) and EG2 (*n* = 29 IUD patients *with* at least one other comorbid mental disorder) to examine the impact of the mental comorbidity on QoL in IUDs (RQ2). The two groups of IUD patients had approximately the same mean age (25.75 vs. 24.34 years, *p* = 0.352), and the distribution of IUD subtypes also showed no significant difference (77.4% video games vs. 75.9% video games, *p* = 0.847). They also did not differ significantly in body mass index (26.8 vs. 27.0 kg/m^2^, *p* = 0.925).

With reference to RQ4, EG1 and EG2 were additionally compared with the CG1 (*n* = 55) and CG2 (*n* = 34). A bootstrapped one-way between-group ANOVA was conducted with the four QoL domains, BAI, BSI, and BDI-II as dependent variables. The ANOVA indicated significant group differences at the *p* < 0.001 level in all QoL domains and in all scales of psychological symptoms (BDI-II, BAI, BSI) regarding the four study groups (effect sizes η^2^ = 0.25–0.49; see Table 3).

**TABLE 3:** Means, standard deviations, and one-way analyses of variance in quality of life (QoL) and comorbidity: comparison of study groups.

Measures	IUD no comorbidity		IUD with comorbidity		Healthy controls		Other mental disorders		*F*(3,145)	η^2^
				
	*n* = 31 (EG1)		*n* = 29 (EG2)		*n* = 55 (CG1)		*n* = 34 (CG2)			
				
	M	SD	M	SD	M	SD	M	SD		
***QoL dimensions***										
Physical health	47.81	11.61	37.68	12.17	61.10	7.36	44.43	14.15	33.74***	.41
Psychological	48.79	12.91	39.79	16.42	69.54	9.06	49.50	15.68	39.52***	.45
Social relationship	46.50	18.10	31.89	23.89	77.12	15.97	53.92	23.23	37.26***	.44
Environment	67.94	12.55	57.54	16.61	84.48	10.08	65.71	20.20	25.33***	.34
***Comorbidity symptoms***										
BDI-II	12.19	8.35	22.45	10.56	1.40	1.58	13.29	10.83	47.42***	.49
BAI	5.77	6.27	14.83	11.26	1.38	1.71	6.86	6.85	25.90***	.35
BSI										
GSI score	0.55	1.30	1.31	0.70	0.09	0.09	0.61	0.65	40.86***	.46
***BSI subscales***										
Somatization	2.00	2.81	5.66	6.08	0.38	0.85	2.35	2.84	16.22***	.25
Obsession-compul.	5.74	4.04	10.52	5.54	1.05	1.32	5.09	5.28	34.45***	.42
Interpers. sensitivity	2.68	2.30	7.48	4.43	0.33	0.74	2.65	3.47	41.39***	.46
Depression	5.16	3.82	11.14	5.21	0.42	0,83	5.32	6.20	42.98***	.47
Anxiety	2.32	2.37	5.48	3.92	0.62	0.95	2.82	3.27	22.06***	.31
Hostility	2.13	2.97	6.28	4.84	0.51	0.79	2.88	3.42	23.06***	.32
Phobic anxiety	1.26	1.51	4.72	4.20	0.25	0.55	1.82	3.56	18.41***	.28
Paranoid ideation	3.00	3.66	6.97	5.18	0.49	0.99	3.18	4.11	21.96***	.31
Psychoticism	2.68	2.93	6.48	4.02	0.18	0.58	2.85	4.19	28.10***	.37

***p < 0.001.

**FIGURE 1:**
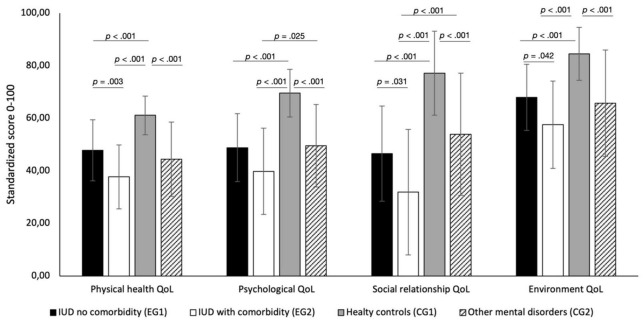
Levels of the 4 quality of life (QoL) dimensions: physical domain, psychological domain, social relations domain, environment domain. Post-hoc tests for the group differences between EG1, EG2, CG1, and CG2 are shown. Only the significant *p* values are shown.

Post-hoc comparison with Bonferroni correction for the QoL domains indicated a constant pattern (see Figure 1) in all QoL domains with mostly significant differences (with the exception of the psychological domain) between EG1 and EG2. H2 could thus be confirmed. Further, we found significant differences in QoL domains between EG1 and CG1, and significant differences between CG1 and CG2. EG1 and CG2 did mostly not differ significantly in all QoL domains. In summary, EG2 showed the lowest score across all QoL domains. Thus, IUD patients *with* comorbid mental disorders (EG2) indicated 20.9% lower QoL compared to IUD patients *without* any comorbid mental disorders (EG1) considering all four QoL domains. In addition, EG2 patients also showed a 21.8% lower QoL compared to CG2 patients. Consequently, EG1 patients showed comparable QoL to CG2 patients. The healthy CG1 reported the highest QoL, as expected. A similar pattern was found in the post-hoc analyses (with Bonferroni correction) for the BDI, BAI, all BSI scales including the GSI (see Table 3, detailed post-hoc data not shown).

### Predictive Power of Internet Use Disorder Symptoms, Psychological Symptoms on Quality of Life

In the bootstrapped correlation analysis for the EG (*n* = 60 IUD patients), highly significant positive associations were found between IUD symptoms (s-IAT score) on the one hand and depressive symptoms [*r*(58) = 0.51, *p* < 0.01], anxiety [*r*(58) = 0.42, *p* < 0.01] and general psychological distress [*r*(58) = 0.52, *p* < 0.01] on the other. Furthermore, significant negative correlations with quality of life were found for the physical domain [*r*(58) = −0.49, *p* < 0.01], the psychological domain [*r*(58) = −0.45, *p* < 0.01], and the environmental domain [*r*(58) = −0.27, *p* < 0.05]. However, when controlling for depression (BDI Score) using partial correlation, the correlations between IUD symptoms, psychological symptoms, and QoL domains mostly lost their significance (data not shown).

Four stepwise multiple regressions were conducted within the IUD sample (*n* = 60) to test which variables can predict each of the four QoL domain (RQ3). The following independent variables were included: IUD symptom severity (s-IAT score), average time spent on the Internet (hours/week), depression symptoms (BDI score), anxiety symptoms (BAI score), global impairment due to psychological symptoms (BSI GSI score), age, partnership (yes vs. no), education (high vs. lower), employment status (no vocational training vs. in training/finished degree/others), the body mass index (BMI), and numbers of mental disorders. The standardized scores for each of the four QoL domains were used as dependent variables.

The stepwise regression for the QoL domain *physical health* indicated that only the BSI score (β = −0.464, *p* < 0.001) and the s-IAT score (β = −0.256, *p* < 0.040) were significant predictors explaining 40.3% of the variance, *R*^2^ = 0.403, *F*(1,56) = 18.55, *p* < 0.001, f^2^ = 0.675. The *psychological* domain of QoL was significantly predicted by only the BDI score (β = −0.850, *p* < 0.001) and a missing of vocational training (β = −0.315, *p* < 0.001), *R*^2^ = 0.675, *F*(1,56) = 57.12, *p* < 0.001, f^2^ = 2.07. In contrast, the QoL domain *social relationship* was significantly predicted by both BDI score (β = −0.354, *p* = 0.006) and time spent on the Internet (β = −0.286, *p* = 0.025), *R*^2^ = 0.289, *F*(1,56) = 11.18, *p* < 0.001, f^2^ = 0.406. Finally, the QoL domain *environment* was significantly predicted only by BDI score (β = −0.611, *p* < 0.001, *R*^2^ = 0.373, *F*(1,56) = 33.35, *p* < 0.001, f*^2^* = 0.594). In summary, H3 had to be partially rejected. The most powerful predictor for low QoL in IUD patients (in the psychological, social, and environmental domain) was the depression symptom severity. IUD factors only showed significantly predictive power in physical QoL (predicted by overall symptom burden and IUD severity) and social QoL (predicted by depressive symptoms and time of Internet use). Employment status (no vocational training) was a significant predictor of psychological QoL while all other socio-demographic variables, the numbers of mental disorders, and the body mass index were non-significant. All regression models reached large effect sizes f^2^ > 0.35.

## Discussion

It has been long debated whether IUDs are addictive disorders in terms of behavioral addictions, or if this concept merely over-pathologizes extensive Internet use and video gaming as an epiphenomenon of other mental disorders (42–45). The results of the present study support the current etiological understanding that IUD (particularly gaming disorder) is an independent mental disorder. IUD showed a discrete impact on QoL even if comorbid disorders have been ruled out using standardized diagnostics (hypothesis 1 confirmed). Furthermore, our study found a consistent QoL pattern: IUD patients showed significant impairments with high effect sizes in all QoL domains. Patients with IUDs had similarly high impairments in QoL as patients with non-IUD (mixed) mental disorders. Both patient groups were recruited at the same hospital, implying that no recruitment bias has to be expected. The combination of an IUD and another comorbid mental disorder decreased the QoL in all four domains by an additional 20% (hypothesis 2 confirmed). Particularly severe impairments in QoL were found in social and psychological QoL. It is known that there is a bidirectional relationship between depression and IUDs (46). Our study shows that the co-occurrence of IUD and depression may lead to a clinical relevance due to the lower levels of QoL, which should be considered a matter of focus in treatment planning.

When switching from the categorical (diagnosis according ICD-11) to the dimensional (degree of symptomatology) perspective, the results of the study showed a more differentiated situation. Contrary to our hypothesis, the regression analyses indicated that the impairment of QoL in psychological, social, and environment domains was mainly predicted by depressive symptoms. In contrast, IUD related factors showed a significant predictive role only in the psychological QoL domain (s-IAT score) and the social QoL domain (time spent on the Internet). The particular impact on the *social* QoL in patients with IUDs is consistent with earlier findings (9, 10) and therapeutic practice, and is therefore also an explicit diagnostic criterion in the DSM-5 (2). The physical QoL was predicted by global burden symptoms (BSI) and IUD severity (s-IAT). This result supports the recent knowledge about the complex association between IUD symptom severity and physical activity, which reports a reciprocal causality between IGD and the level of sport/exercise (47). The body mass index did not affect quality of life in our IUD sample. However, previous studies indicate that increased screen time and Internet use correlate with higher body weight and obesity (48, 49). Overall, the healthy group had the lowest BMI, the IUD patients and the patients with other mental disorders did not differ in BMI here. Thus, our study could not show any specific effect of media use on body weight.

The s-IAT score was mainly not a significant predictor of QoL when including depression symptoms in the regression models. These results were supported by the partial correlation between QoL domains and IUD symptomatology. Thus, when controlling for BDI score, all positive correlations between s-IAT, psychological symptoms, and QoL domains lost significance. In summary, hypothesis 3 had to be rejected in part. These findings may suggest that the association between IUD symptom severity and QoL could be meditated by depressive symptoms. A further mediation analysis on a sufficiently large sample could examine this more closely. On the other hand, the lower effect of IUD addiction symptoms on QoL could be explained by the clinical experience that many IUD patients feel a pleasurable relationship with video gaming and Internet usage. Video gaming, for example, is often experienced as self-esteem stabilizing and enjoyable, and thus often functions as an ultimate coping strategy against symptoms of depression (15). Therefore, IUD patients could underrate their problematic Internet use (s-IAT score), which could explain a loss of significance in the regression analyses. Many affected individuals are reluctant to give up computer gaming, but rather suffer from the social consequences and accompanying depressive symptoms. If they do not get “pressure” from their social environment (e.g., failed education, pressure from parents, unemployment), they would often describe life in the virtual world as (at least temporarily) very worthwhile and pleasurable. Therefore, further studies could assess the severity of IUD symptoms by patients and by their parents. Recent studies of adolescents with IGD and their parents suggested differences in assessment between affected subjects and their parents (50, 51).

The frequency of anxiety disorders was higher in the IUD group than in the patient control group. It cannot be excluded that the higher number of anxiety disorders could have an impact on quality of life. However, this is contradicted by our finding that anxiety symptoms (BAI) and number of mental disorders were not significant predictors in the regression.

The effect of depressive symptoms on QoL in IUD might have a clinical consequence. If depressive symptoms are relevant for the QoL in IUD patients, the integration of anti-depressive therapy strategies into IUD treatment could be considered to improve QoL. This might apply to psychopharmacological treatment, although evidence of efficacy is not strong (52, 53). A recent systematic review reported the antidepressants bupropion and escitalopram as successfully tested in some clinical studies (53). CBT-oriented IUD psychotherapy, on the other hand, show good efficacy in IUD patients, and already includes many anti-depressive therapeutic elements, e.g., activity planning and implementation, cognitive restructuring (54, 55).

### Limitations

The study is a cross-sectional study. Therefore, causal inferences can only be considered with caution. In addition, the sample sizes are limited, although the power analysis indicated a sufficient sample size. The strength of the study is that clinical patients were examined using standardized clinical interviews by experienced clinicians. However, multivariate analyses were calculated using self-assessment instruments. A further limitation is that only male IUD patients were studied and therefore also compared with only male control groups. The transmission to female IUD patients is therefore not possible, especially since women often use other Internet applications.

## Conclusion and Further Directions

Male IUD patients showed a significantly lower QoL compared to age-matched healthy men supporting the clinical relevance of IUD. Furthermore, IUD patients *with* additional comorbid mental disorders even reported lower QoL than comparable non-IUD patients with other mental disorders. Depressive symptoms were a significant predictor of low QoL in IUD patients. Strategies to reduce depressive symptoms should therefore be considered in the treatment of IUD patients to improve QoL. Further IUD studies should examine QoL using self-assessment and additional assessments from relatives/partners, and should use mediation analysis. The physical and social QoL were most impaired und should be addressed more detailed. IUD patients often lose touch with their bodies, become overweight or do not exercise enough. This physical component should be further investigated, as many current therapeutic approaches do not yet include the stimulation of physicality.

## Data Availability Statement

The original contributions presented in the study are included in the article/supplementary material, further inquiries can be directed to the corresponding author.

## Ethics Statement

The studies involving human participants were reviewed and approved by Ethics Committee of the Ruhr-University Bochum (no. 15-5155). The patients/participants provided their written informed consent to participate in this study.

## Author Contributions

JD-H did the main conceptualization, conducted the statistical analysis, did the interpretation of the data, and wrote the first draft of the manuscript. BT and TS supported the conceptualization, designed and supervised the data collection, and did critical review, commentary, and revision. MP, LB, HK, and SH supported the interpretation of data and did critical review, commentary, and revision. All authors have read and approved the manuscript.

## Conflict of Interest

The authors declare that the research was conducted in the absence of any commercial or financial relationships that could be construed as a potential conflict of interest.

## Publisher’s Note

All claims expressed in this article are solely those of the authors and do not necessarily represent those of their affiliated organizations, or those of the publisher, the editors and the reviewers. Any product that may be evaluated in this article, or claim that may be made by its manufacturer, is not guaranteed or endorsed by the publisher.
